# Clinical Remarks on a Peculiar Case of Defective Utterance, under the Care of P. Martin Duncan, M. D., F. G. S., Physician to the Essex and Colchester Hospital

**Published:** 1850-07

**Authors:** 


### ARTICLE IV.

*Clinical Remarks on a peculiar case of Defective Utterance, under the care of* P. MARTIN DUNCAN, M. D., F. G. S., *Physician to the Essex and Colchester Hospital.*

CASE. Lucy Halls, a tall, well-made, married woman, twenty-six years of age, became one of my patients in the Essex and Colchester Hospital on March 25th, 1849. She has had the usual diseases of childhood. Her catamenia appeared when she was fourteen years of age. She married two years ago, and has had no children, nor is she now pregnant. There is no insanity in her family. Her head is well formed; and she is generally quick and intelligent. She has always lived well and regularly. Has been tolerably free from mental anxiety, and has never had hysterical symptoms of any kind. There is a cicatrix on the left trochanter, and she remembers the existence of an abscess there many years ago. Has never had fits during her infancy, and indeed has enjoyed good health up to a few weeks since, when her appetite failed her, and her menstrual flux diminished in quantity and color. After awhile she complained of nausea, headache, and occasionally of giddiness; she became pallid, and after these symptoms had persisted for fourteen days, she suddenly fell down senseless, and remained so for a few minutes. She was not convulsed; her face was flushed, and on her recovery she suffered from severe frontal headache. In the course of a day or two, the headache still persisting, she had another fit, lost her consciousness as before, and was not convulsed, but on recovering found that her speech was affected in a peculiar manner. A

third fit of like character enhanced the last-mentioned symptom, and she then came under my care.

March 25, 1849.—Her general appearance is good; she is upright and stout; has not, nor has she had, any skin-disease; countenance depressed and rather anxious; her lips are pale, and she has the aspect of slight general anæmia; she complains of constant intense frontal headache, more acute on the left than on the right side; of dizziness, and of a sensation of overpowering debility; her headache prevents her from sleeping, and is increased by the horizontal posture; she has neither tenderness of scalp nor facial paralysis; the tongue is protruded, retracted, and moved in every direction with the greatest facility, but whenever she attempts to utter a syllable, however simple, its tip is forcibly directed against the fore part of the palate, is retained there but for an instant, and as rapidly returns; her language is, therefore, a succession of "d's" and vowels. "Yes, sir," is rendered "ded, dud." "Pain in my head, sir," is made into "did, did, did, ded, dud;" one word following readily, and not too rapidly, after the other. "Hospital" is "dod, did, dud," in her method of pronunciation; and any polysyllable is a jumble of d's and vowels. She can pronounce neither gutturals nor sibilants. The word "why," which requires the use of the lips only, she can enunciate tolerably well, but it is frequently called "wid;" indeed there is a tendency on her part to end all syllables by a "d," or "t." She tries to pronounce correctly, and cries bitterly after her unsuccessful attempts. She has neither startings, nor paralysis of any of the muscles of the limbs or trunk; her senses are intact; she has never suffered from globus hystericus, or from spinal tenderness; nor can any structural changes be distinguished about the tongue, jaws, or neck; she has neither leucorrhœa nor pain in the side; heart and lungs healthy; urine scanty and highly lithic; appetite none; bowels constipated; tongue flabby and foul; no thirst; no pain after eating; pulse 96, full and excitable. Ordered low diet. Blister to left temple. R. Pil. Hydrarg., gr. v., omni nocte. Haust. Sennæ bis.

26th.—Better; headache less; bowels moved; articulation the same; pulse 96. Pergat.

27th.—Headache nearly gone; articulation worse, and as she is loud-voiced and loquacious, can be heard—"did, dod, dud, did," and so on—all over the ward.

28th.—Much the same. Pergat.

30th.—Headache has left her, the appetite is returning, the bowels are well open, the urine is no longer loaded with lithates, she is paler than she was when she was admitted, and suffers from slight nervous palpitation. Middle diet. R. Ferri Sulph., gr. ij; Ammon. Carb., gr. v.; Mist. Gent. Co., oz. j., ter die.

April 7th.—During the past week her general appearance has improved, her appetite is still not overgood, she no longer complains of headache, but her speech is no better. R. Quinæ Sulph., gr. ij; Inf. Rosæ, oz. j., ter die.

8th.—Slept badly. R. Pil. Saponis Co., gr. v., hac nocte.—Rep. Haust. ter die.

11th.—Is better in every respect, her speech alone excepted; bowels slightly constipated; has excited herself a good deal on account of her not being able to make herself understood by her friends, and is slightly hysterical. R. Pil. Hydrarg., gr. iij.; Ext. Col. Co., gr. v. ft. pil. ij., hâc nocte sumendæ. R. Tinct. Assafœtid., dr. ss.; Tinct. Valerian. Co., dr. ss; Ferri Sulph., gr. ij.; Mist. Camph., oz. j., ter die.

13th.—Much the same; hysterical symptoms gone; speech not improved; is still pale; tongue clean; bowels open; pulse 84. R. Ferri Sulph., gr. ij.; Mist. Camph., oz. j., ter die.

May 8th.—No improvement since the last report; articulation the same; is stronger, and has more color. R. Strych., gr. 1-10th; Sp. Vin., dr. j.; Mist. Camph., oz. j., ter die.

9th.—Much the same. Pergat.

12th.—Articulation decidedly improved; she can occasionally pronounce a sibilant sound. Strych., gr. ⅛th; Sp. Vin., dr. ij.; Mist. Camph., oz. j., ter die.

14th.—Speech much improved; she can say "yes, sir, why, and could," much better, but after awhile returns to the "did dud." Pergat.

17th.—Improved greatly; catamenial flux more profuse; appetite good; spirits excellent; still some impediment in her speech. Pergat.

22nd.—Speaks fluently, continuously, and without the least impediment. Omit Med. She is now in good health, and has perfect speech.

Remarks.—In this case there evidently was no aphonia, the chordæ vocales accomplished their functions in a perfect manner, as did also the muscles of the oral sphincter. The tongue was the offending member, that delicate approximation of its tip to the forepart of the palate, which is requisite for the production of sibilant sounds, no longer existed; a violent, sudden and ill-directed effort, propelled the tongue in the proper direction, but the want of the power of adaptation prevented the production of the wished-for sound. Words beginning with a hard "c or k," as "could, cork, kiss," which require the back part of the tongue to be impinged for an instant against the corresponding part of the palate, could not be pronounced by my patient; she had not power whilst speaking to elevate the back part of the tongue; on the contrary, the above-mentioned words were rendered "dud, dod, did," the top of the tongue being employed, yet, when not speaking, she could move the tongue in all directions. Speaking in a whisper was accompanied by the above-mentioned odd ill adaptation of the muscular effort.

In order to have perfect voice and perfect enunciation, we require a perfect co-adaptation and co-ordination of function of the muscles of expiration—of the chordæ vocales—muscles of the soft palate—muscles of the tongue, and of those muscles of the face which have to do with the oral opening.

In this case the functions of all the structures were perfect, with the exception of those of the muscles of the tongue; there was a want of the power which controls and directs the muscular efforts of the tongue during the act of speaking; yet all was well when the tongue was to be propelled or retracted, without speech being attempted.

Now, the tongue is protruded by the combined action of the genio-hyo-glossi; its top is propelled against the fore-part of the palate, by the same muscles, assisted in a certain degree by the linguales; it is retracted from its elongated condition by some of the fibres of the genio-hyo-glossus of each side, by the stylo-glossi, and when the os hyoides is fixed, by the linguales. When the tongue is altogether within the cavity of the mouth, the genio-hyo-glossi have nothing to do with retractation; this is left to the stylo-glossi, assisted occasionally by the linguales. The ninth nerves supply the genio-hyo-glossi and linguales muscles; the stylo-glossi are supplied by branches of the glosso-pharyngeal nerves.

The peculiarities of this case were excess of action of the propelling muscles, and a want of power in the retractor muscles of the tongue; most likely the first condition was an effect of the second,—paralysis of one set, and necessary (for their antagonistic force was lost) increased action of the other set of muscles. But this was only the case during speech; there were no evidences of paralysis of any of the muscles of the tongue at any other time; on the contrary, paralysis of the stylo-glossi can only be the result of lesion, either at the origin, or during the course, of the glosso-pharyngeal nerves. Can we imagine such a paralysis or such a lesion to exist at one time and not at another?—not to exist during ordinary attempts at exertion, and to exist when the action required was more or less automatic and co-existent with a combination of several functions. Can paralysis of a muscle exist during an effort of speech, and not at any other time, or during any other occupation? It did in this case, and I believe the following to be the correct explanation:—A certain amount of nervous energy is required for the production of the usual muscular efforts of propulsion, retractation, and rotation of the tip of the tongue; also for the impinging the back part of the organ against the palate; but a much greater amount is required to regulate the delicate movements employed during the act of speech. A nerve injured to a certain extent will produce one series of phenomena, but has not, or cannot transmit, as the case may be, influence enough to produce a more complex and exacting series.

To produce a muscular effort, a certain amount of grey globular nerve matter is necessary to originate, and a perfect system of nerve tubes to conduct, the requisite nervous force, the more complete the adaptation of the originating with the conducting nervous structures, the more complete will be the powers of the muscle to which they bear relation. Every collection of nerve fibres has a certain definite number or quantity of the globular nervous element in relation with it, and the loss of any portion of this last will render the nervous influence of a certain number of fibres weaker, they will have less nervous force to conduct; they may be able to stimulate a muscle to certain exertions, but not to others, requiring more nervous energy.

The branches of the glosso-pharyngeal nerves which supplied the stylo-glossi muscles in this case, were able to stimulate them to certain, but not to special and more exacting efforts, on account of a loss of the proper quantity of grey matter usually existing at their roots or points of origin. How came this loss? The patient had three fits after suffering from some considerable constitutional disturbance; the first fit resembled what is termed fugitive apoplexy, the second and third, resembled the first in character, but were more severe, and left behind them that lesion of the brain which prevented the glosso-pharyngeal nerves from influencing the stylo-glossi in a proper and sufficient manner. The after symptoms were not sufficiently severe to lead one to believe that a clot ever existed. I feel inclined to refer the fits and the slight structural change to one of these local congestions, which are by no means unfrequent in other organs besides the brain in women, whose catamenia are not as they should be, and who are more or less anæmic. Sufficient injury could be done by the long-continued pressure of dilated capillaries upon the nervous substance within their meshes, to account for the loss of the usual and sufficient power.

I was led to believe from the commencement of the case that it was not of an inflammatory kind, and that some localized congestion alone existed. Purgatives relieved the most distressing of the head symptoms; and the tonics, which were subsequently administered, although they increased the general tone of her health, yet had no influence upon the affection of the speech. Strychnia was then administered, and she soon felt the beneficial effect of it; the dose was increased, and she rapidly regained the controlling power over her tongue. She suffered from no startlings whilst she was taking the strychnia, which, in her case, as I believe in all others, acted upon that part of the brain which had suffered more or less structural change.

We notice that in cases of hemiplegia, depending upon non-inflammatory softening of the brain, or following upon an apoplexy, (the clot being supposed absorbed,) if strychnia be exhibited, the starting produced by it are seen in the paralyzed limbs first; the drug appears to fix its action upon the diseased, rather than upon the normal structures. It is the greatest restorer of nervous energy we possess.—*Prov. Med. and Surg. Jour.*

